# Supplementary material for: Martini 3 Model of Cellulose Microfibrils: On the Route to Capture Large Conformational Changes of Polysaccharides
Source: Molecules. 2022 Feb 1;27(3):976. doi: 10.3390/molecules27030976 (PMC8838816; doi:10.3390/molecules27030976)
Supplement: Supplementary file 1 [file molecules-27-00976-s001.zip › molecules-1545298-SI.pdf]

Supplementary Material: Martini 3 Model of Cellulose Microfibrils: On the Route to Capture Large Conformational Changes of Polysaccharides

Rodrigo A. Moreira, Stefan A.L. Weber and Adolfo B. Poma

1. Structure of the coarse-grained (CG) D-glucose molecules

Below we show the intra-residue angle CG1-CG3-CG2 and the inter-residue angle CG6-CG3-CG2 along the trajectory for all chains and CG fibrils (i.e. Iα, Iβ and type-II). See Figure 2 in main text for definition of CG beads. The results are shown in Figure 1, where we can see that distributions on angles are well defined. Moreover, the intra-residue distribution is nearly the same for all three fibrils ($\sim$30.8 degrees), as expected by the construction of the model. However, the inter-chain distributions for the fibril type-II shows an angle distribution with a tail closer to 180 degrees than in Iα and Iβ cases. This feature characterises a more linear structure for type-II, and consequently a less twisted fibril, exactly as described in Table 4 of the main text.

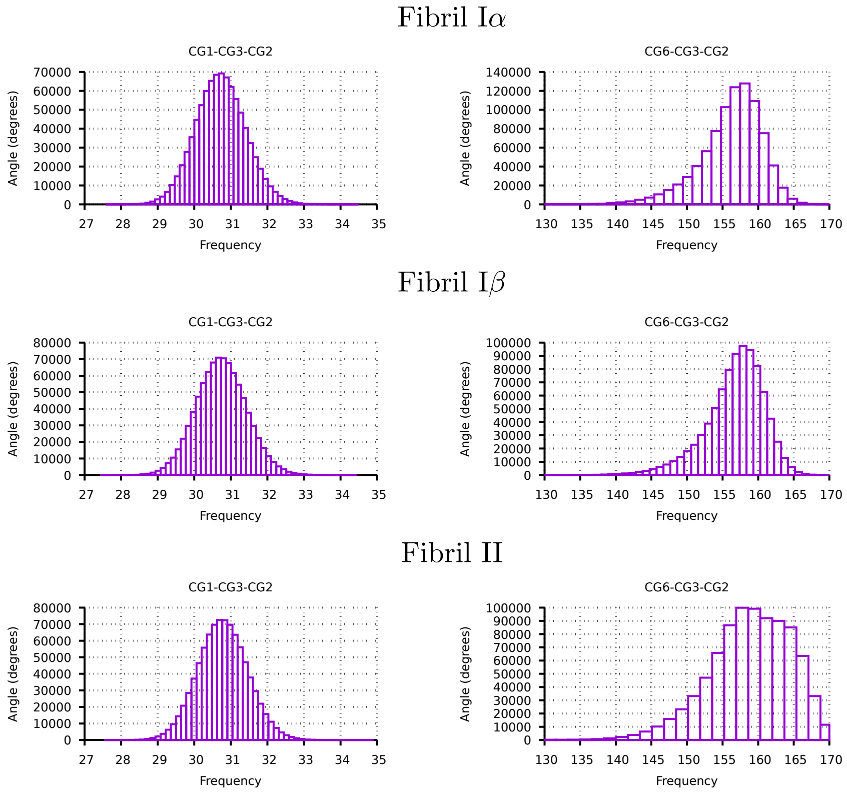

Figure S1: Histogram of intra and inter residues angles from CG dynamics.

2. CG mapping scheme

Generally, the 4:1 mapping scheme in Martini model is not a necessary constraint in the approach. In principle, it guides the CG parametrization on how to use the Martini 3 library. Previous implementations of the Martini model for cellulose fibrils, which were limited to describing one cellulose type (e.g. generally Iβ), the typical 4:1 scheme provides 3 CG beads per D-glucose. In our study, we have employed a different mapping scheme using a mathematical construction defined by triangular patches that optimally cover an entire surface of the D-glucose. This process is mathematical robust and includes one additional CG bead, as

in total 4 CG beads, and in combination with Martini 3 model is capable to capture more systems with one unique parametrization.

3. Thermal stability of the CG model

Our model is robust over a range of temperature. Thus, we performed an exhaustive thermal analysis for model validation. The solvated systems were annealed in temperature over 100ns and 1bar, as implemented by GROMACS 2020.4, namely:

- 0ns $\rightarrow$ 25ns at 300K
- 25ns $\rightarrow$ 50ns from 300K to 400K : linear increase of temperature
- 50ns $\rightarrow$ 100ns at 400K

Below we show the dynamics of RMSD computed using as a reference the first frame of the annealing procedure, and the average radius of gyration (R_{GYR}) computed from each cross-section of the fibril (defined by the same residue number of each fibril). The R_{GYR}

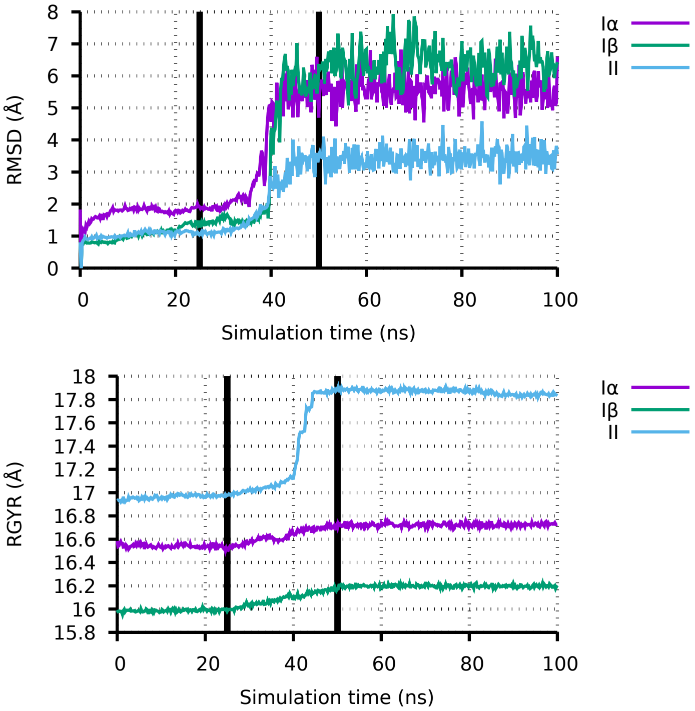

Figure S2: Annealing results for Iα, Iβ, type-II cellulose fibrils.

variation is at most $\sim$5%, which reflects the conservation of the cross-section of the fibril, even after 100K increase in temperature, and for a timescale of about 50ns. The RMSD also corroborates the stability of the fibrils, as it is stable along the same time scale. Note that comparing the fibril RMSD using a reference frame equilibrated at 300K is not an optimal choice, but it is enough to asses the relative structural relaxation. This results show that our model is thermally stable above physiological temperatures. More details on the fibril dynamics at high temperatures will be the focus on a subsequent study.
